# Prostate cancer addiction to oxidative stress defines sensitivity to anti-tumor neutrophils

**DOI:** 10.1007/s10585-022-10170-x

**Published:** 2022-05-23

**Authors:** Diane L. Costanzo-Garvey, Adam J. Case, Gabrielle F. Watson, Massar Alsamraae, Arpita Chatterjee, Rebecca E. Oberley-Deegan, Samikshan Dutta, Maher Y. Abdalla, Tammy Kielian, Merry L. Lindsey, Leah M. Cook

**Affiliations:** 1 Department of Pathology and Microbiology, University of Nebraska Medical Center, 985900 Nebraska Med Center, Omaha, NE 68198 USA; 2 Department of Psychiatry and Behavioral Sciences, Texas A&M College of Medicine, Bryan, TX USA; 3 Department of Medical Physiology, Texas A&M College of Medicine, Bryan, TX USA; 4 Department of Cellular and Integrative Physiology, University of Nebraska Medical Center and Omaha VA Medical Center, Omaha, NE USA; 5 Department of Biochemistry & Molecular Biology, University of Nebraska Medical Center, Omaha, NE USA

**Keywords:** Metastasis, Neutrophils, Prostate cancer, Bone, Reactive oxygen species, Metabolism

## Abstract

Bone metastatic prostate cancer (BM-PCa) remains one of the most difficult cancers to treat due to the complex interactions of cancer and stromal cells. We previously showed that bone marrow neutrophils elicit an anti-tumor immune response against BM-PCa. Further, we demonstrated that BM-PCa induces neutrophil oxidative burst, which has previously been identified to promote primary tumor growth of other cancers, and a goal of this study was to define the importance of neutrophil oxidative burst in BM-PCa. To do this, we first examined the impact of depletion of reactive oxygen species (ROS), via systemic deletion of the main source of ROS in phagocytes, NADPH oxidase (Nox)2, which we found to suppress prostate tumor growth in bone. Further, using pharmacologic ROS inhibitors and Nox2-null neutrophils, we found that ROS depletion specifically suppresses growth of androgen-insensitive prostate cancer cells. Upon closer examination using bulk RNA sequencing analysis, we identified that metastatic prostate cancer induces neutrophil transcriptomic changes that activates pathways associated with response to oxidative stress. In tandem, prostate cancer cells resist neutrophil anti-tumor response via extracellular (i.e., regulation of neutrophils) and intracellular alterations of glutathione synthesis, the most potent cellular antioxidant. These findings demonstrate that BM-PCa thrive under oxidative stress conditions and such that regulation of ROS and glutathione programming could be leveraged for targeting of BM-PCa progression.

**Supplementary Information:**

The online version contains supplementary material available at 10.1007/s10585-022-10170-x.

## Introduction

Cancer metastasis significantly reduces patient survival [1]. Although a fairly inefficient process, a critical component of metastatic growth involves deconstruction and modification of specific tissue microenvironments to facilitate tumor growth. This phenomenon is particularly relevant for bone metastatic cancers, including prostate cancer, which metastasizes to bone more frequently than other tissue site [2]. The bone is a nutrient rich environment, regulated by bone-modeling osteoclasts and osteoblasts, which drive bone osteolysis and osteogenesis, respectively. Metastatic cancer cells specifically activate both bone remodeling populations, benefiting from the release of sequestered bone growth factors from resorbed bone [3], creating a “vicious cycle” of tumor growth in bone [4]. Despite the extensive evidence of prostate tumor interactions within the bone cavity, specific bone-targeted therapies have been unsuccessful in improving patient survival requiring a need for other therapeutic options for patients with bone metastatic disease [5].

Although bone stromal cells have been characterized for decades as to their role in cancer progression, the bone environment is predominantly comprised of hematopoietic stem cells and myeloid precursors, notably precursors of neutrophils [6, 7]. Based on the abundance of neutrophils in bone and their known contributions to cancer progression [8], we previously investigated the role of neutrophils in the prostate tumor-bone microenvironment. In contrast to a number of studies identifying pro-tumoral neutrophils in prostate cancer [9–11], we found that bone marrow neutrophils are initially protective against prostate tumor growth in bone in a STAT5-dependent manner, such that STAT5-positive prostate cancer cells are targeted for neutrophil killing [12]. These studies define tumor intrinsic properties that dictate neutrophil immune response against prostate cancer. Further, we found that prostate cancer cells induce neutrophil functions classically defined as bactericidal mechanisms [12]; however it is unclear how or whether those tumor-induced changes regulate prostate tumor progression. In this study, we focused on the mechanism of tumor-induced neutrophil function in the tumor-bone microenvironment.

Neutrophils utilize a number of mechanisms to target bacterial pathogens, including production of reactive oxygen species (ROS), or oxidative burst, which is largely regulated by the action of membrane-associated enzyme, NADPH oxidase 2 (NOX2; also known as cytochrome b subunit beta or cytochrome b-245 heavy chain (CYBB)) [13]. NOX2 generates superoxide by transferring electrons from NADPH to oxygen; the accumulation of intra- and extracellular ROS contributes to oxidative stress and as a result, death of pathogens and mammalian cells [14, 15]. Thus, maintenance of balanced ROS production with antioxidant activity is critical for cell viability. Because of heightened metabolic needs, tumor cells adapt to chronic oxidative stress associated with progression to aggressive and metastatic disease [16, 17]. Previous studies have identified altered neutrophil redox metabolism to contribute to tumor progression of other cancers [16, 18]; however, there is no evidence of the role or impact of neutrophil metabolism in prostate cancer or in the prostate tumor-bone environment.

In this report, we demonstrate that prostate cancer cells induce neutrophil oxidative burst, with metastatic prostate cell lines inducing abundant amounts of neutrophil intracellular ROS, compared to non-metastatic prostate cancer and non-malignant prostate epithelial cells. Increased ROS in the tumor microenvironment could either directly promote tumor growth via cell damage, or indirectly promote tumor growth through inhibition of cytotoxic lymphocytes. The role of extracellular ROS in the prostate tumor-bone environment, and particularly when derived from neutrophils, remains unclear. Here, we demonstrated that metastatic prostate cancer cells promote neutrophil ROS production and suppress neutrophil antioxidant function, thereby maintaining cancer oxidative stress conditions. Additionally, prostate cancer cells reduce neutrophil glutathione metabolism further promoting extracellular ROS accumulation. Inhibition of neutrophil ROS in vitro significantly inhibited growth of AR-negative prostate cancer cells and sensitized them to neutrophil killing, suggesting a disease stage-specific response to oxidative stress. Likewise, in vivo inhibition of NOX2, significantly inhibited metastatic prostate cancer growth in bone. Our findings reveal that metastatic prostate cancer regulates neutrophil redox metabolism to maintain heightened ROS levels in the microenvironment, which has unveiled a novel mechanism for targeting aggressive, therapy resistant metastatic prostate cancer.

## Materials and methods

### Cell culture and reagents

Cell lines were cultured under the following conditions: PC3, C42B, RM1, PAIII- DMEM (Hyclone SH30243.01), 10% FBS (PEAK PS-FB1), 1% penicillin/streptomycin (P/S) (Gibco 15140-122) and LNCaP- RPMI (Hyclone SH30027.02), 10% FBS, 1% P/S. ROS inhibitors: Apocynin 100uM (Millipore 498-02-2), gp91 ds-tat 10uM (Anaspec AS-63818), sgp91 10uM (Anaspec 63821), phorbol 12-myristate 13-acetate (PMA) 100 nM (Caymen 10008014), Rotenone 5uM (Sigma 13995), N-acetyl-l-cysteine (NAC) 5 mM (Sigma 1009005) and Diphenyleneiodonium chloride (DPI) (Selleckchem S8639) and 100 μM Butathione Sulfoximine (BSO) (Caymen 14484). All drugs were used according to manufacturers’ instructions. Prostate cancer conditioned media (CM) was collected as previously described in serum free RPMI [12].

### Mouse studies

Because this study is focused on prostate cancer, only male mice were used. For neutrophil isolations, C57BL/6 mice were purchased from Jackson Laboratory. Mice between 9and12 weeks of age were used for all experiments using primary neutrophils. *Nox2* knockout *(KO)* mice (B6.129S-*Cybb*^*tm1Din*^/J) and age-matched C57Bl/6J mice, with *Nox2 WT* expression, were a gift from Tammy Kielian (University of Nebraska Medical Center;UNMC). Mice were housed on a 12 h light/dark cycle, with free access to food and water. All procedures performed were approved by IACUC (UNMC).

### In vivo experiments

Male *Nox2*^*wt*^ (n = 4–5) and *Nox2*^*−/−*^ (n = 4–5) mice, ages 7–9 weeks were used for intratibial injections of luciferase-expressing RM1 cells, and performed twice. Approximately, 4.5 × 10^4^ luciferase-expressing RM1 cells were injected into the left tibia or saline was injected in the contralateral limb as a control. Tumor burden was longitudinally tracked using IVIS bioluminescence imaging (Perkin Elmer). Data shown is from all mice from both experiments. D-luciferin (GoldBio) was given at 150 mg/kg intraperitoneally, and images captured 15 min after injection. Images were acquired every two days, and data reported as relative luminescent intensity (RLU). Tumor burden per mouse was normalized to the RLU at Day 1 post injection to control for variability in mouse tumor take. For ex vivo analyses, wildtype Nox2 mice (n = 3), and Nox2^−/−^ mice (n = 2), representative of median tumor burden, were used to collect data from tumor-associated neutrophils (TANs). Remaining tumor limbs from each group were used for downstream histological analyses. As controls, neutrophils were isolated from non-tumor bearing mice intratibially injected with saline tumor naiive, were used as controls. TANs and tumor naïve neutrophils were isolated from bone marrow of tibia using Mojosort negative selection (Biolegend 480,057). ROS production was measured via Amplex Red, and killing capacity measured via co-culture. For tumor osteolysis, radiographic images (Faxitron X-ray Corp) were obtained using an energy of 35kVp and an exposure time of 8 ms. Osteolysis was measured as a readout of tumor volume (TuV) and calculated as a function of the total tissue volume (TV).

### In vivo ROS measurement

For detection of in vivo ROS, L-012 (Fuji 120-04891) luminescent reagent (40 mg/kg) was injected intraperitoneal and luminescence was measured via IVIS every minute for 10 min. Luminescence was quantified in tumor bearing limbs vs. non-tumor bearing limbs.

### Neutrophil isolation from bone marrow

Neutrophils were isolated from bone marrow of C57BL/6 male mice, or *Nox2*^*wt*^ and *Nox2*^*−/−*^ mice. Bone marrow was flushed from femur and tibia of each mouse, using a 25 gauge needle with approximately 500 μl of isolation buffer (PBS, 2% FBS and EDTA) per bone into 1 mL Eppendorf tubes. Bones were filtered with a 70 μm filter, to remove bone fragments. Bone marrow from each mouse was counted and neutrophils were isolated using the negative selection MojoSort mouse neutrophil isolation kit. Manufacturers’ instructions were followed exactly as written.

### Dihydrorhodamine 1,2,3 (DHR) assay

For measurement of intracellular ROS, DHR was used. Neutrophils were isolated from mouse bone marrow of *Nox2*^*wt*^ and *Nox2*^*−/−*^* mice*, using the MojoSort isolation kit, as described above, and (1 × 10^5^) cells per gentotype, in triplicate, were pre-incubated in RPMI media with either NAC (5 mM), gp91-ds-tat (10 μM) or DPI (10 μM) for 30 min at 37 degrees. After incubation, neutrophils were washed with 1X PBS to remove inhibitors, then resuspended in PBS with 5 μM DHR 123 (ThermoFisher D23806), and PMA (100 nM) added as a positive control. 100 μl of cells were added to 96 well black plate and fluorescence was measured at 488 nm, using Tecan plate reader.

### MitoSox assay

Mouse bone marrow neutrophils were isolated from *Nox2*^*wt*^ and *Nox2*^*−/−*^ mice and 5 × 10^4^ neutrophils were plated in triplicate per condition in a black, clear bottom 96 well plate in RPMI media. RPMI was supplemented with PMA (100 nM), rotenone (5 μM), or PMA and rotenone in combination. For analysis of mitochondrial ROS (mROS), 5 μM MitoSox reagent (ThermoFisher M36008) was added to each well. After a 30-min incubation, fluorescence was measured using a Tecan plate reader at 510 nm. For experiments using tumor-associated neutrophils (TANs), only basal levels of mROS were assayed with no additional treatment added.

### Co-culture assay

Direct co-culture procedures were followed as previously described [12]. PCa cells were plated, in triplicate, 24 h prior to the addition of neutrophils, in either 24 or 48 well plate. Neutrophils were added the following day at 10:1 ratio of neutrophils to cancer cells. Neutrophils were allowed contact with cancer cells overnight (~ 18–24 h), media removed, and viability of cancer cells assessed using Trypan Blue exclusion assay. For co-cultures where neutrophils were treated with inhibitors: neutrophils were washed with PBS following isolation, counted, and resuspended in base media with or without NAC (5 mM) or apocynin (100 μM) and placed at 37 degrees for ~ 1 h. Following one wash with PBS to remove the inhibitors, neutrophils were placed on the cancer cells, for the remainder of the overnight incubation.

### Amplex Red assay

For analysis of extracellular secretion of the abundant ROS, H_2_O_2_, we used the Amplex Red assay (ThermoFisher A12222). For measurement of neutrophil ROS, 3 × 10^5^ mouse neutrophils were treated with LNCaP, C42B, PC3, and RM1 CM for 4 h. After CM treatment, neutrophils were washed and re-suspended in 1X PBS, and ~ 1 × 10^5^ cells plated per well of a 96-well plate in triplicate per condition. Amplex red buffer with horse radish peroxidase (HRP) was added to the cells, and incubated at room temp in the dark, according to assay protocol. Fluorescence measurements were read at indicated time-points at 530 nm using Tecan plate reader.

For analysis of neutrophil ROS after co-culture, LNCaP, C42B, PC3, or RM1 cells were plated in triplicate in a 24 well plate. Mouse neutrophils were added to the culture after prostate cells were attached (~ 18–24 h later), at 10:1 neutrophils to cancer cells. Neutrophils were removed from co-culture with cancer cells after 3 h of incubation at 37 degrees, washed, counted and 50,000 neutrophils re-suspended in 50 μl 1X PBS, and placed in a black 96 well plate (Corning 3904), with each condition performed in triplicate. Amplex Red buffer with HRP was added to begin the reaction. Cells were incubated in the dark at room temp for the indicated time-points, and fluorescence read at 530 nm. Concentrations were calculated using a hydrogen peroxide (H_2_O_2_) standard curve.

### Measurement of total glutathione

Analysis of glutathione was performed using GSH/GSSG-Glo Assay (Promega, V6611), a luminescent assay for measuring total glutathione. Neutrophils (2 × 10^5^) isolated from bone marrow were treated with prostate cancer CM for ~ 3 h, washed in PBS, and plated in a white 96 well plate, with ~ 6–7 × 10^4^ cells per well in triplicate. Luciferin reagent was added and luminescence was measured using a Tecan plate reader, after allowing 20 min to equilibrate. GSH and GSSG were quantified given the formulas recommended by the manufacturer. To perform the assay on cultured cancer cells and from co-culture, neutrophils were removed from the cancer cells, and reagents applied directly to cancer cells. The same protocol reagents were used, but volume increased for use on adherent cells, per manufacturers’ instructions. After the first lysis step, cell lysates were transferred to a white 96 well plate to complete the remainder of the assay. For glutathione inhibitor assays, cancer cells were treated with BSO (100 μM) for 24 h. BSO was then removed from the cancer cells and neutrophils were added at 10:1 ratio to cancer cells. Counting of the viable cancer cells was done as previously described using trypan blue exclusion ~ 24 h after neutrophils were added.

### RNA sequencing of human bone marrow neutrophils

Human bone marrow was purchased from Lonza and neutrophils were isolated using a modified Ficoll gradiant centrifugation protocol, as described [12]. Isolated neutrophils were treated with either LNCaP or C42B CM (n = 3 replicates per CM) for 3 h and RNA isolated using Trizol (Invitrogen). RNA sequencing (RNA-seq) was performed on 6 total human neutrophil samples treated with CM. Samples were analyzed with respect to purity and potential degradation in the UNMC Genomics Core Facility. Purity and concentration were assessed by measurement of the A260/280 ratios using a Nanodrop (Thermo Scientific, Nanodrop Products, Wilmington, DE) instrument and samples with values of 1.8 to 2.0 were processed. Potential degradation of the sample was assessed by analysis of 200 ng of the RNA with an Advanced Analytical Technical Instruments Fragment Analyzer (AATI, Ames, IA) and only intact RNA samples were utilized to create sequencing libraries. Libraries were generated using 1 μg of total RNA from each sample and the TruSeq V2 RNA sequencing library kit from Illumina following recommended procedures (Illumina Inc., San Diego, CA). Libraries were multiplexed and sequenced on the NextSeq500 DNA Analyzer (Illumina) to generate a total of approximately 20 to 25 million 75 bp paired reads for each sample. FASTQ files were provided to the Bioinformatics and Systems Biology core for further analysis. *Bioinformatics.* The original FASTQ format reads were trimmed by fqtrim tool (https://ccb.jhu.edu/software/fqtrim) to remove adapters, terminal unknown bases (Ns) and low quality 3’ regions (Phred score < 30). For quality control (QC), the low quality 3′ regions were trimmed and only high quality reads with Phred score ≥ 30 were retained. The trimmed fastq files were processed by newly developed (in the UNMC Bioinformatics Core) standard pipelines utilizing STAR [19] as the aligner and RSEM [20] as the tool for annotation and quantification at both gene and isoform levels. The RNAseq reads were mapped to the reference genome, quantified and annotated through the gtf file. The gene numbers were determined by the gtf genomic annotation file. Further bioinformatics was performed using Ingenuity Pathway Analysis software (IPA; Qiagen) and Gene Ontology (GO) analyses. Additional gene set analysis was performed using the ENRICHR program. The data discussed in this publication have been deposited in NCBI's Gene Expression Omnibus and are accessible through GEO Series accession number GSE193468.

### Western blot

Protein was quantified using the Bio-Rad DC Protein Assay Kit (BioRad 5000111) according to manufacturer instructions. 50 μg of each protein sample was loaded per well of a 12% SDS gel and then transferred to Immobilon-P PVDF membranes. Primary antibodies HMOX1 (Enzo, ADI-SPA-896-F), Catalase (Abcam, ab76024), Mn-SOD (Millipore, 06-984), Cu–Zn-SOD (Abcam, ab51254), and Beta Actin (Cell Signaling Tech, 4970) were diluted 1:1000 in 5% milk in TBST and incubated overnight at 4C on a rocking platform. HRP-conjugated secondary antibody (goat-anti-rabbit IgG, Enzo, ADI-SAB-300) was diluted 1:5000 in 5% milk in TBST. Azure Biosystems Radiance Plus was added to each blot according to the manufacturer’s instructions and blots were imaged on an Azure c600.

### Statistical analyses

Sample size was chosen based on a confidence level of 95% with a 5% margin of error. *p*-value < 0.05 was considered as statistically significant. Error bars represent standard error from the mean (SEM). All statistical analyses were performed with Graph Pad Prism 6.0 (Graphpad Inc., LaJolla, CA). Bioinformatics was performed by the UNMC Bioinformatics and Systems Biology Core.

## Results

### Impact of reactive oxygen species on BM-PCa growth.

**Fig. 1:**
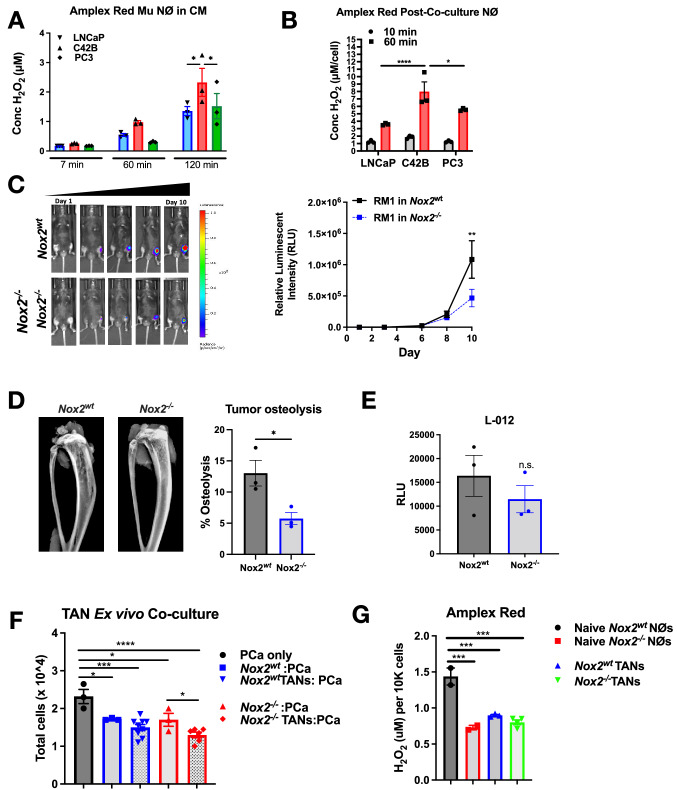
The role of ROS in BM-PCa progression. **A** Hydrogen peroxide (H_2_O_2_) detection via Amplex Red assay in primary bone-derived mouse neutrophils incubated 3 h with LNCaP, C42B or PC3 CM. Graph shows micromolar (μm) concentration of H_2_O_2_ per well. **B** H_2_O_2_ detection via Amplex Red assay from neutrophils after direct co-culture with prostate cancer cells. Neutrophils were removed from co-culture and washed with PBS after 5 h of contact with cancer cells. Neutrophils were then resuspended in PBS with the addition of amplex red reagent, and secreted H_2_O_2_ detection monitored using the Tecan plate reader; n = 3 for each cell line per experiment. Graph shows concentration of H_2_O_2_ per well. Data are represented as mean ± SEM. Statistical analysis per two way ANOVA with p-values as follows: *p < 0.05, ****p < 0.0001. **C** Representative bioluminescent images of tumor burden (left) and quantitation (right) of relative luminescent intensity after normalizing luminescence to Day 1 post injection of tumor cells. **D** Faxitron X-ray images (left) and quantitation (right) measuring percent tumor-induced bone osteolysis in tibia marrow, using ImageJ software to quantify osteolysis. **E** Quantitation of L-012, a bioluminescence indicator of ROS, relative luminescent intensity (RLU) to tumor burden per mouse; measurement was taken one day prior to the end of study. n = 3 mice per group. **F** Tumor naïve or TANs were isolated from mouse tibia using negative selection (MojoSort). Ex vivo assays were plated in triplicates per mouse. Direct co-culture of tumor naiive and TANs with RM1-luc-RFP cells incubated overnight (~ 16 h). After incubation, cancer cell number was assessed via trypan blue exclusion. Graph shows remaining cancer cell number. (G) Amplex Red assay of basal extracellular ROS levels in TANs isolated from tumor study. Cells were incubated in PBS with amplex red reagent for 30 min and secreted H_2_O_2_ detection monitored using the Tecan plate reader. Data are represented as mean ± SEM. Statistical analysis per one ANOVA with p-values as follows: *p < 0.05, **p < 0.01, ***p < 0.001, ****p < 0.0001

We previously identified bone marrow neutrophils elicit an anti-tumor immune response in vitro and protect against metastatic prostate cancer growth in bone. In vitro, we identified that prostate cancers induce neutrophil activation identified through enhanced NET formation and intracellular reactive oxygen species (ROS) production, i.e., oxidative burst. Based on these findings, we examined neutrophil secreted ROS after induction by prostate cancer cells and specifically wanted to examine differences in neutrophil ROS production induced by metastatic (C42B, PC3) compared to non-metastatic prostate cancer (LNCaP) [20–23]. NADPH oxidase 2 (NOX2) generates superoxide in neutrophils, which is rapidly converted by antioxidants, such as superoxide disumutase (SOD), to the less labile, non-radical hydrogen peroxide H_2_O_2_. Primary mouse bone marrow-derived neutrophils were treated with prostate conditioned media (CM) for 3 h, whereupon excess CM was removed, and extracellular H_2_O_2_ was measured using Amplex Red, which detects extracellular H_2_O_2_; Amplex® Red reagent in a 1:1 ratio in the presence of horseradish peroxidase (HRP) produces the red fluorescent oxidation product resorufin, that can be measured fluorometrically and the amount of H_2_O_2_ released can be calculated by comparison to a standard curve. There was more H_2_O_2_ detected in C42B-treated neutrophils as early as 7 min of detection, compared to LNCaP-treated neutrophils (Fig. 1A). For comparison to another BM-PCa cell, we treated neutrophils with CM from PC3 cells, which were isolated from a bone metastasis of prostate cancer and are androgen independent, representing the most aggressive stage of bone metastatic prostate cancer [24]. Although PC3-treated neutrophils had released the least amount of H_2_O_2_ by 60 min of detection (0.30 μM), at 2 h there were nearly equal amounts of H_2_O_2_ released by LNCaP- and PC3-treated neutrophils (1.35 and 1.52 μM H_2_O_2_, respectively) (Fig. 1A).

Based on our previous findings demonstrating contact-dependent neutrophil immune responses [12], we next examined ROS secretion after direct contact with prostate cancer cells. To do this, bone marrow-derived neutrophils were cultured in direct contact with LNCaP, C42B and PC3 cells in vitro at a 10:1 ratio with cancer cells, based on our previous studies. After 4 h, neutrophils were removed from culture and Amplex Red was added to neutrophils ROS measurement at 10 and 60 min, using the same procedure as stated above. Similar to CM treatment, direct culture induced extracellular ROS secretion from neutrophils. At ten minutes, there was at ~ 1–2 uM H_2_O_2_ secreted from neutrophils cultured with each prostate cancer cell line. By 60 min of H_2_O_2_ detection, C42B-cultured neutrophils had produced the most H_2_O_2_ (8.0 μM ± 2.2) with similar levels produced by neutrophils cultured with PC3(5.6 μM ± 0.178)_._ Notedly, neutrophils cultured with bone metastatic PCa (C42B and PC3 cells), secreted significantly more H_2_O_2_ (~ 2.5 fold) than neutrophils cultured with non-metastatic LNCaP (3.6 μM ± 0.14) revealing that direct contact between neutrophils and prostate cancer cells results in heightened neutrophil oxidative burst, even more so than induction by CM (Fig. 1B). These data collectively demonstrate that PCa induces neutrophil ROS production and this is further enhanced with cell–cell contact between neutrophils and prostate cancer cells.

Excess accumulation of intracellular and extracellular ROS can induce cellular oxidative stress, which can promote cell death of non-malignant cells. However, malignant cells of various tumor types, exhibit heightened ROS production and flourish under oxidative stress conditions, suggesting an addiction to oxidative stress associated with cancer progression. To investigate the importance of ROS on BM-PCa progression, we interrogated growth of bone metastatic prostate cancer in the nicotinamide adenine dinucleotide phosphate (NADPH) oxidase 2 knockout (*Nox2*^*−/−*^) mouse model. Nox2 involves ATP and is the predominant Nox isoform expressed in phagocytic cells [25]. Loss of Nox2 has been associated with impaired innate immune response and, specifically an inability of neutrophil anti-bacterial response [26–28]. Luciferase-expressing mouse prostate cancer cells, RM1, which were derived from the mouse Ras-Myc oncogene prostate model [29], were injected intra-tibially in male *Nox2*^*wt*^ and *Nox2*^*−/−*^ mice (n = 9/group), which exhibit homozygous *Nox2* expression. Bone tumor growth was measured longitudinally via bioluminescence imaging. In comparison to *Nox2*^*wt*^*,* RM1 tumor burden was significantly reduced by 57% (*p* < 0.01) in *Nox2*^*−/−*^ mice by the end of the study, at Day 10 (Fig. 1C). Similarly, there was significantly less cancer-induced bone osteolysis in *Nox2*^*−/−*^ knockout mice (Fig. 1D). To determine ROS within the tumor tibia, L-012, a luminescent probe that produces light when ROS is detected was given to mice (n = 3/group). Luminescence was quantified via IVIS imaging, indicating relative amount of ROS present. There was reduced L-012 luminescence in *Nox2*^*−/−*^ tumor-bearing limbs, though not significantly, suggesting that there was still residual ROS in the tumor-bone microenvironment, likely from the tumor cells or host cellular mitochondrial ROS (Fig. 1E).

To examine the role of tumor-associated neutrophil (TAN) cytotoxicity ex vivo, TANs were isolated from the tumor-bearing tibia of RM1 from Nox2-wildtype (n = 3) and -null male mice (n = 2), for comparison to neutrophils from tumor naiive mice. There were fewer bone marrow cells and TANs in wildtype Nox2 tumor bones compared to *Nox2*^*−/−*^ mice (Supp. Figure 1A), likely due to the amount of tumor in the bone marrow of wildtype mice compared to Nox2-null. Wildtype Nox2- and Nox2-null TANs, and tumor-naïve neutrophils, were co-cultured with RM1 cells at 10:1 ratio overnight and the remaining RM1 were counted the following day, using trypan blue exclusion, as previously described [12]. As previously seen, all bone marrow neutrophils induced significant cancer cell death ex vivo, specifically inducing ~ 26% RM1 cell death by *Nox2*^*wt*^ tumor-naïve neutrophils (*p* < 0.05) and ~ 36% death by *Nox2*^*wt*^ TANs (*p* < 0.05). *Nox2*-null TANs showed a significantly heightened killing capacity (~ 44% RM1 cell death; *p* < 0.0001),compared to tumor naïve *Nox2*-null neutrophils (~ 27% RM1 cell death; *p* < 0.05) (Fig. 1F). However, there was little difference in TAN cytotoxicity when comparing Nox2 wildtype vs. Nox2-null TANs.

Neutrophils produce ROS via Nox-dependent and Nox-independent (via the mitochondria) mechanisms. As seen previously, tumor-naïve Nox2-null neutrophils produced significantly less H_2_O_2_ than tumor naïve wildtype Nox2 neutrophils (*p* < 0.001), as detected using the Amplex Red assay. Surprisingly, wildtype Nox2 TANs produced less than the tumor naïve wildtype Nox2 neutrophils and produced similar H_2_O_2_ to Nox2-null cells. Next, we measured TAN mitochondrial ROS using MitoSox. MitoSox detects mitochondrial specific ROS. It permeates live cells where it rapidly oxidized by superoxide, producing a red fluorescence. Tumor-naïve *Nox2*^*−/−*^ neutrophils produced ~ 12% less basal mitochondrial ROS than wildtype Nox2 neutrophils and, likewise, *Nox2*^*−/−*^ TANs produce significantly less (23%; *p* < 0.0005) mitochondrial ROS than *Nox2*^*wt*^ TANs (Supp Fig. 1B). There was little difference in mitochondrial ROS when comparing Nox2-null TANs with tumor-naïve neutrophils indicating that mitochondrial superoxide levels are not changed due to the presence of tumor in the microenvironment. These data collectively suggest that Nox2 is important for PCa growth in the bone environment and that prostate cancer cells may benefit from neutrophil oxidative burst.

### Importance of Nox2 and ROS in neutrophil cytotoxicity

**Fig. 2:**
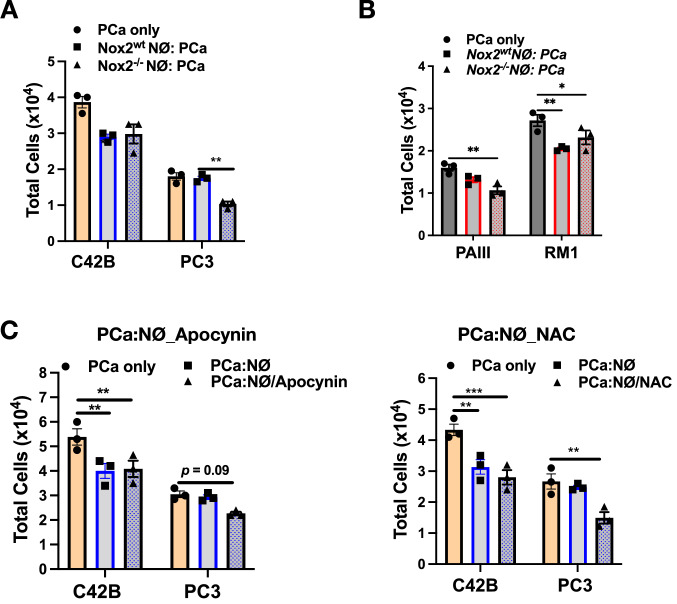
Impact of pharmacological and genetic inhibition of neutrophil Nox2 on neutrophil-induced prostate cancer cell death. **A** C42B and PC3 or **B** RM1 and PAIII cancer cells cultured overnight with primary bone-marrow derived mouse wildtype Nox2 (*Nox2*^*wt*^) and Nox2-null (*Nox2*^*−/−*^) neutrophils, in a 10:1 ratio of neutrophils to cancer cells. Graphs represent total cells remaining after trypan blue exclusion. **C** C42B and PC3 cancer cells cultured overnight with primary bone-marrow derived mouse WT neutrophils that were pre-treated for ~ 60 min with apocynin (100 μm) or **D** NAC (5 mM), inhibitor removed, and neutrophils added directly to cancer cells overnight (16 h). Graphs represent total cancer cells remaining after trypan blue exclusion; n = 3 per cell line, per experiment. Data are represented as mean ± SEM. Statistical analysis per two way ANOVA with p-values as follows: *p < 0.05, **p < 0.01, ***p < 0.001

We previously showed that bone marrow neutrophils induce cell death of prostate cancer cells, using in vitro co-culture assays. To more specifically investigate the role of neutrophil ROS in prostate cancer growth, we utilized Nox-2 deficient neutrophils in prostate cancer co-culture assays, where primary wildtype or Nox2-null neutrophils were cultured with PCa cells overnight and PCa cell numbers counted using Trypan Blue exclusion assay. Bone marrow derived neutrophils from *Nox2*^*wt*^ and *Nox2*^*−/−*^ mice were co cultured with C42B, PC3 and RM1 cancer cells at 10:1 ratio of neutrophils to cancer cells. Both wildtype and Nox2-null neutrophils induced C42B cells death; however, *Nox2*^*−/−*^ neutrophils induced PC3 cell death (~ 40% cell loss, *p* < 0.01) in comparison to *Nox2*^*wt*^ neutrophils (Fig. 2A). We previously showed that PC3 cells are resistant to neutrophil killing and our findings here suggest that loss of Nox2, i.e. neutrophil ROS, induces PC3 death. Likewise, neutrophils induced RM1 cell death although there was little difference in wildtype vs. Nox2-null neutrophil cytotoxicity despite significant RM1 growth suppression seen in Nox2 knockout mice (Fig. 2B). One key difference between C42B, PC3 and RM1 cells, aside from species difference, is that PC3 androgen-receptor (AR) negative and represent castration-resistant PCa. Performing a co-culture of neutrophils and cancer cells, we next examined neutrophil killing of AR-negative PAIII cells, a rat prostate adenocarcinoma cell line which is bone metastatic in vivo. As seen with the other cell lines, wildtype neutrophils induced PAIII cell death; similar to PC3, *Nox2* knockout neutrophils showed enhanced killing of PAIII cells (Fig. 2B).

Dihydrorhodamine 123 is a nonfluorescent reactive oxygen species (ROS) indicator that can passively diffuse across membranes where it is oxidized to rhodamine 123 which exhibits a green fluorescence in presence of intracellular ROS. To determine if intracellular ROS was affected in *Nox2*^*−/−*^ neutrophils, *Nox2*^*wt*^ and *Nox2*^*−/−*^ neutrophils were incubated in RPMI with DHR dye and PMA, a potent NADPH oxidase activator, as positive control. *Nox2*^*−/−*^ neutrophils produce significantly less intracellular ROS compared to wildtype neutrophils (Supp Fig. 2A), and are unable to be stimulated with PMA. These results were validated by treating wildtype neutrophils with gp-91-ds-tat, a small peptide inhibitor of Nox2, and DPI, a pan-inhibited of all Nox enzymes. There was a 37% reduction in fluorescence of PMA-stimulated neutrophils treated with Nox2 inhibitor gp91-ds-tat (*p* < 0.0001), and a 60% reduction (*p* < 0.0001) in DPI-treated neutrophils compared to PMA-stimulated neutrophils not treated with inhibitors (Supp Fig. 2B), demonstrating that Nox2 inhibition reduces intracellular neutrophil ROS. Likewise, Amplex Red assay revealed significantly less extracellular ROS production by *Nox2*^*−/−*^ neutrophils (5.9 μM ± 1.3) compared to wildtype (8.2 μM ± 0.18) and an inability of Nox2-mediated extracellular ROS production upon PMA stimulation (Supp Fig. 2C). However, there was still some baseline ROS produced by non-PMA-stimulated ROS. MitoSox assay showed that there was no difference in neutrophil mitochondrial ROS in the absence of Nox2 and, using the complex I inhibitor Rotenone, we found that both wildtype and Nox2-knockout neutrophil mitochondrial ROS was stimulated (Supp Fig. 2D). These findings demonstrate that Nox2 depletion significantly reduces intra- and extracellular ROS but has little impact on mitochondrial ROS production.

We next wanted to determine whether the findings from co-culture assays (Fig. 2A and 2B) were dependent on Nox2 depletion rather than ROS. To do this, we inhibited neutrophil ROS by treatment with either apocynin or N-acetyl-L-cysteine (NAC) prior to culture with PCa cells. Apocynin, a pan-NOX inhibitor, blocks serine phosphorylation of p47 subunit and assembly with gp91 complex at the membrane [30]. NAC, a synthetic precursor of cysteine and glutathione, acts to promote scavenging of ROS [31]. Neutrophils were treated with apocynin or NAC for 30 min and directly cultured with C42B and PC3 cells. We found that apocynin- and NAC-treated neutrophils depleted of ROS were to induce C42B cell death (Fig. 2B), comparable to vehicle control-treated neutrophils. Interestingly, similar to culture with Nox2-null neutrophils, depletion of neutrophil ROS sensitized PC3 cells to neutrophil killing (Fig. 2B). Collectively, these findings demonstrate that inhibition of neutrophilic ROS induces death of AR-negative (PC3, PAIII), with little impact on AR-positive (C42B, RM1), castration-resistant metastatic prostate cancer.

### BM-PCa regulation of neutrophil transcriptome

**Fig. 3:**
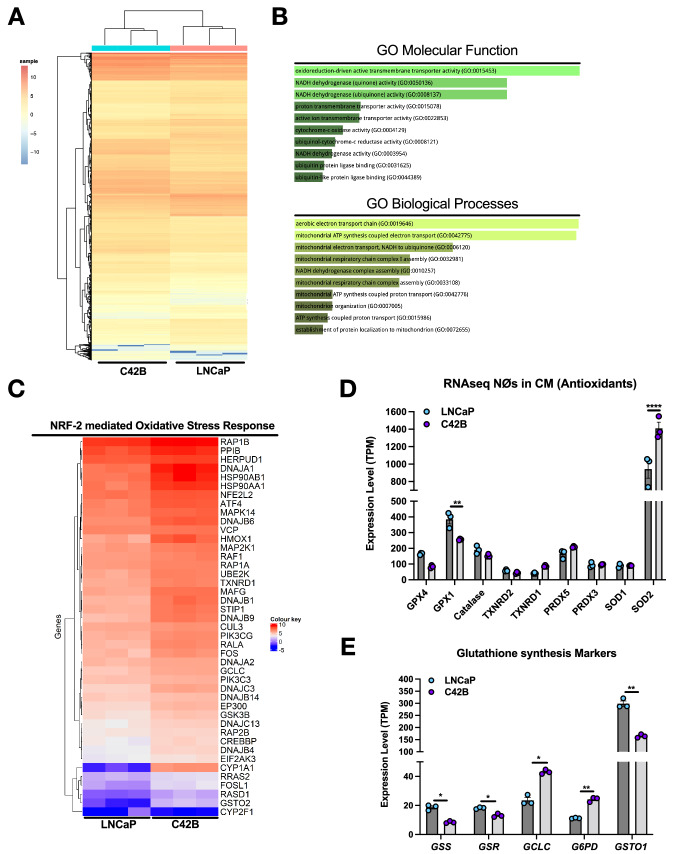
BM-PCa regulation of the transcriptome of bone marrow-derived neutrophils. **A** Heatmap of significantly regulated neutrophil genes. Color key; red shows increased gene expression per fold-change, blue represents reduced gene expression fold-change. **B** Gene ontology (GO) analysis of molecular function and biological processes. **C** Heatmap from RNA sequencing analysis of human neutrophils treated with non-metastatic LNCaP or metastatic C42B CM; specific genes are from the NRF-2 mediated Oxidative Stress Response pathway as identified by IPA secondary analysis. Color key; red shows increased gene expression per fold-change, blue represents reduced gene expression fold-change. **D** Antioxidant enzymes or **E** glutathione specific markers from RNA sequencing data. Graphs show expression levels as transcripts per million (TPM); n = 3 per prostate cancer CM. Data are represented as mean ± SEM. Statistical analysis per two-way ANOVA with p-values as follows: *p < 0.05, **p < 0.01, ****p < 0.0001

Our data suggest that prostate cancer induction of neutrophil ROS may assist with cancer growth maintenance and, ultimately, tumor progression. This is based on the fact that depletion of neutrophil ROS, negatively impacts growth of specific prostate cancer subtypes. To gain insight into the molecular changes associated with BM-PCa-induced neutrophil oxidative burst, we performed bulk RNA sequencing on human bone marrow neutrophils treated with prostate cancer conditioned media (CM) from non-metastatic LNCaP cells or bone metastatic C42B cells for 3 h. Approximately 57,000 genes were analyzed (full gene list can be found at GEO Accession: GSE193468): 3318 genes were down-regulated > twofold; 836 genes were up-regulated > twofold. This gene list was further analyzed by Ingenuity Pathway Analysis (IPA) software. Approximately 32,000 genes mapped to IPA and there were 477 genes significantly either up- or down-regulated greater than 1.5-fold (false discovery rate < 0.05); (Fig. 3A). The top most activated canonical pathways in C42B-treated neutrophils, compared to LNCaP-treated neutrophils, were: unfolded protein response (21 of 55 genes, including several heat shock protein family members (*HSPA2, HSPA8*) and activating transcription factor(*ATF*) 4), hypoxia signaling in the cardiovascular system (23 of 72 genes, including genes associated with the *UPR*, *HSP90AB1* and *ATF4*), and NRF-2-mediated oxidative stress response (39 of 187 genes, including glutathione S-transferase omega 2 (*GSTO2*, tenfold increase) and heme oxygenase 1 (*HMOX1*, fourfold increase)). Other pathways identified to be associated with significant gene modifications, included: IL-6 signaling, TGFβ signaling, and PI3K/AKT signaling, though there was overlap in many genes over several different pathways mapped to IPA libraries (Supp Fig. 1B, pathway connectivity map). Although it is unclear what regulates these observed changes, the top two most predicted upstream pathway mediators, based on gene mapping to IPA libraries, were hormone receptor signaling mediators (Table 1): nuclear receptor subfamily 3 group C member 1/glucocorticoid receptor (NR3C1/GCR; *p* = 4.11E−10) which is homologous to the androgen receptor, and can propagate androgen-mediated signaling, and estrogen receptor 1 (ESR1; *p* = 3.77E−6) [32]. Further, Myc proto-oncogene (n-Myc/c-Myc) was identified as the 3rd most predicted upstream pathway regulator, and has been notably associated with prostate cancer progression [33–35].

**Table 1:** Top ten predicted upstream regulators of neutrophil gene regulation in response to prostate cancer CM treatment

Upstream regulator	Expr false discovery rate (q-value)	p-value of overlap	Target molecules in dataset
*NR3C1*	6.38E−01	4.11E−10	ANXA1, ARID4B, ATG4B, ATP1B1, ATXN1, BAG2, BAG3, BAG4, CARD14, CARD6
*ESR1*	3.92E−01	3.77E−06	ABCC5, ABLIM1, BAZ2A, C18orf25, CCL20, CD55, CDKN1A, CNOT4, CRKL, CXCL3
*MYC*	6.51E−01	1.02E−05	CCND2, CCT3, CDKN1A, CSDE1, CTDSP1, DLEU1, EIF4E, EIF4G1, ENO1, FOSL1
*SATB1*	4.63E−02	1.81E−05	CDKN1A, CEACAM1, CLEC2B, EVI2A, GLRX, GPR18, HSP90AA1, HSPA8, ILF3, LRRN3
*FOXO4*	2.99E−01	2.85E−05	BNIP3, CCN2, CDC42EP3, CDKN1A, JAG1, LEMD3, PSMD11, SGK1
*TP53*	2.45E−01	4.20E−05	ACSL3, AEN, AHSA1, ANXA1, AQP3, ARL6IP1, ATF3, BIK, BNIP3, CCN2
*SMAD4*	3.52E−02	4.82E−05	CAB39, CCL20, CCN2, CDC42EP3, CDK17, CDKN1A, CITED2, DAXX, JAG1, LEMD3
*ESRRG*		6.29E−05	CDKN1A, ENO1, GAPDH, HK2, LDHA, PKM, TPI1
*ELF4*	6.17E−01	1.04E−04	CA2, CREM, CXCL2, CXCL8, PIK3C3, RAP1B, RCAN1
*CEBPA*	9.98E−01	1.14E−04	ANXA1, ARG1, BCL2A1, BIK, CA2, CCND2, CD3G, CDKN1A, CXCL8, CXCR4

Ingenuity Pathway Analysis predicted upstream regulators of neutrophils treated with C42B compared to LNCaP CM. Table shows upstream regulator, associated statistical power and downstream target molecules altered within the RNAseq dataset

There was also significant regulation of genes associated with a heightened pro-inflammatory and suppressed anti-inflammatory immune response, including increased interleukin 8 (*IL8*; 7.5-fold, *p* < 0.01), increased *IL1 alpha* and *IL1R* (~ threefold and fourfold respectively, *p* < 0.05) and increased *IL6R* (twofold; *p* < 0.05) along with a reduction in *IL10R* expression (2.5 fold, *p* < 0.01) (Supp Fig. 3; GEO Accession: GSE193468). These changes have also found to be associated with increased ROS production via priming of neutrophils for oxidative burst, another notable phenotype of pro-inflammatory and activated neutrophils [36–38]. Further, gene ontology analysis of molecular processes and functions of the genes significantly regulated > twofold in C42B-treated (compared to LNCaP) neutrophils revealed the most altered function and processes to be: oxidoreduction-driven active transmembrane transporter activity and aerobic electron transport chain, respectively (Fig. 3B). This suggests altered redox metabolism in neutrophils treated with the secreted factors from bone metastatic PCa.

We next decided to more thoroughly interrogate our RNA sequencing data to examine other molecules associated with oxidative stress that are altered in cancer-treated neutrophils. Pathway analysis of the RNA sequencing data revealed a significant enhancement of the Nuclear factor erythroid 2-related factor 2 (NFE2L2/NRF2)-mediated oxidative stress pathway in C42B-treated neutrophils compared to LNCaP-treated neutrophils (Fig. 3C). NRF2 is a master regulator of genes that have antioxidant response elements and drives the production of antioxidants in response to oxidative stress. Notably, there was an abundance of increase in antioxidant genes such as CYP1A1, and several heatshock family protein members indicative of a stress response. Additionally, we examined changes in other genes that affect oxidative stress. Interestingly, C42B soluble factors reduced expression of antioxidant enzymes such as glutathione peroxidase (GPX)-1 (*p* < 0.01) and -4 suggesting an imbalance of ROS in neutrophils incubated in metastatic PCa CM. GPXs catalyze the conversion of peroxide to water and oxygen, thereby acting to reduce oxidative stress from intracellular H2O2 accumulation. Additionally, there was a significant increase in superoxide dismutase 2 (SOD2), the mitochondrial enzyme that converts superoxide to hydrogen peroxide (Fig. 3D). Similar to increased H_2_O_2_ production (Fig. 1A), these data suggest that C42B promotes H_2_O_2_ conversion from superoxide but suppresses its further conversion.

Glutathione (GSH), is the most potent antioxidant or detoxifier in cells, is responsible for maintaining redox status, as well as many cellular processes, including gene expression, DNA and protein synthesis, cell proliferation and apoptosis, and immune response [39, 40]. RNAseq of bone marrow neutrophils revealed an increase in expression of enzymes involved in glutathione metabolism/synthesis in neutrophils treated with C42B CM, compared to LNCaP media (Fig. 3E). Specifically, detoxifying enzymes including glutathione disulfide reductase (GSR), and glutathione s-transferase omega-1 (GSTO1) were significantly reduced (*p* < 0.05), suggesting that oxidant scavenging is altered. Glutamate—cysteine ligase catalytic subunit (GCLC), the first rate-limiting step of glutathione synthesis, was increased by ~ 84% in neutrophils treated with C42B CM (*p* < 0.05). Although glucose-6-phosphate dehydrogenase (G6PD), which is necessary for the reduction of GSH, was increased (*p* < 0.01), glutathione synthetase (GSS), the second enzyme in the glutathione biosynthesis pathway, was decreased (*p* < 0.05). These findings collectively suggest that metastatic C42B augments ROS-regulating enzymes as well as the major antioxidant system, glutathione production, which may dramatically alter neutrophil redox metabolism and ROS in the tumor-bone microenvironment.

### The role of glutathione metabolism in neutrophils and BM-PCa growth

**Fig. 4:**
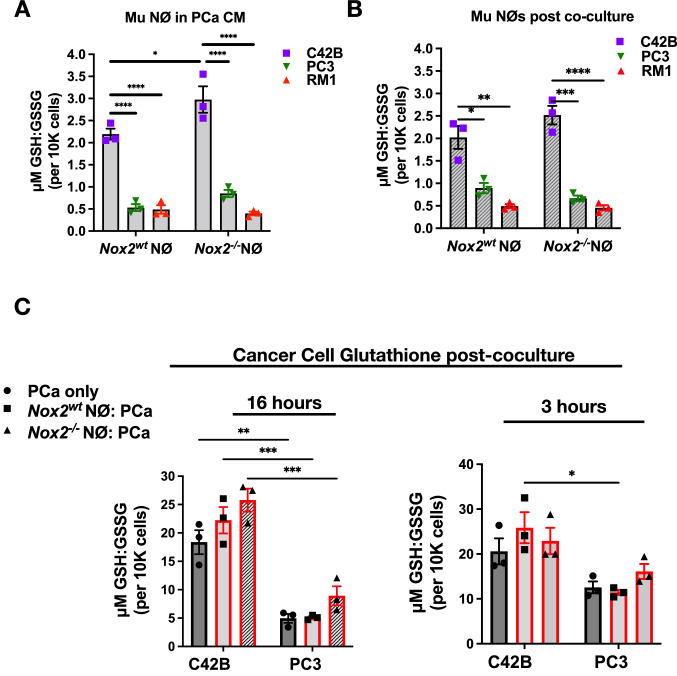
Prostate cancer induces changes in neutrophil glutathione metabolism. **A** Glutathione assay of *Nox2*^*wt*^ and *Nox2*^*−/−*^ neutrophils treated with prostate cancer CM for 3 h. Neutrophils were rinsed and resuspended in PBS, and added to a 96 well plate with glutathione reagents to detect reduced glutathione (GSH) and oxidized glutathione (GSSG). **B** Glutathione assay of neutrophils after 3 h direct culture with prostate cancer cells. Neutrophils were removed from cancer cells, rinsed and resuspended in PBS and assay performed as stated above. Graphs show ratio of reduced glutathione (GSH) to oxidated glutathione (GSSG) as a measure of overall glutathione metabolism. **C** Glutathione assay of C42B and PC3 cells after culture with neutrophils for 16 h (left) and 3 h (right). Neutrophils were removed from co-culture and remaining cancer cells were incubated in PBS with glutathione assay reagents. Graphs show ratio of reduced glutathione (GSH) to oxidated glutathione (GSSG) as a measure of overall glutathione metabolism. n = 3 replicates per cell line. Data are represented as mean ± SEM. Statistical analysis per two way ANOVA with p-values as follows: *p < 0.05,** *p* < 0.01,***p < 0.001, ****p < 0.0001

Our findings collectively show dysregulation of enzyme expression that governs synthesis of the potent antioxidant glutathione. Based on these data, we next examined the amount of glutathione (reduced GSH compared to oxidized GSSG) in cancer CM-treated neutrophils as a readout glutathione metabolism. GSH levels in cells exist 98% in reduced form, with the oxidized molecule only comprising a small portion of intracellular GSH. The ratio of reduced GSH:oxidized GSSG has been shown to be an indicator of oxidative stress [41]. Using the Promega GSH/GSSG glo assay, both total glutathione and GSSG determinations are based on the reaction where GSH-dependent conversion of a GSH probe, Luciferin-NT, to luciferin by a glutathione-S-transferase enzyme is coupled to a firefly luciferase reaction. Light produced is proportional to the amount of GSH present. Determining the total glutathione and amount of GSSG are performed in separate reactions. One configuration determines reduced GSH, the second, the oxidized form, GSSG. This assay is performed directly in a white 96 well plate. To measure the GSH:GSSG ratio in neutrophils, mouse bone marrow neutrophils were treated with CM from C42B and, for comparison, PC3 and mouse RM1 prostate cancer cells. Additionally, we compared wildtype, *Nox2*^*wt*^, and *Nox2*^*−/−*^ neutrophils in this assay. All cells in these assays were confirmed to be viable prior to the assay measurements. C42B CM increased GSH:GSSG in *Nox2*^*−/−*^ neutrophils over wildtype Nox2 neutrophils (~ 30%; *p* < 0.05) (Fig. 4A). The most striking results were with neutrophils treated with PC3 and RM1. For both *Nox2*^*wt*^ and *Nox2*^*−/−*^ neutrophils, PC3 CM significantly reduced GSH:GSSG levels, (in wildtype-76% reduction, in *Nox2*^*−/−*^ -71% reduction (*p* < 0.0001), compared to C42B-treated neutrophils). RM1 media produced similar results as PC3 and significantly reduced neutrophil GSH:GSSG levels (Fig. 4A). For comparison, we examined GSH:GSSG production in neutrophils after culture with prostate cancer cells. As seen with CM treatment, both wildtype and Nox2-null neutrophils cultured with PC3 and RM1 cells produced significantly less GSH compared to culture with C42B cells (Fig. 4B). These data reveal that aggressive metastatic PCa (e.g., PC3 and RM1) would induce a significant amount of oxidative stress in neutrophils by increasing ROS along with simultaneous suppression of oxidant scavenging via GSH production.

Typically, GSH can exist at a 100:1 ratio in non-malignant epithelial cells to the oxidized GSSG, however this ratio is substantially reduced in cancer cells with high ROS levels and exist in oxidative stress. To determine how neutrophil oxidative stress might affect the prostate cancer cells, we examined GSH:GSSG ratios of cancer cells after culture with neutrophils. To do this, we cultured C42B and PC3 cells with primary bone marrow neutrophils overnight (~ 16 h), then measured the ratio of GSH:GSSG in the cancer cells after removal of remaining neutrophils. There was approximately fourfold more basal level GSH:GSSG in C42B cells compared to PC3 cells (*p* < 0.01), and this was based predominantly on the amount of reduced GSH in C42B (Fig. 4C). With the addition of wildtype Nox2 neutrophils into culture, there was a slight but non-significant increase in GSH of C42B compared to C42B alone (~ 18% increase; *p* = 0.08). This increase was further enhanced with the addition of *Nox2*^*−/−*^ neutrophils onto C42B. Despite our findings demonstrating that *Nox2*^*−/−*^ neutrophils induce C42B apoptosis similarly to wildtype Nox 2 neutrophils (Fig. 2A), C42B cells still respond to “ROS-reduced” neutrophils by regulating intracellular GSH:GSSG. There was no change in PC3 glutathione levels with the addition of wildtype Nox2 neutrophils; however the addition of *Nox*^*−/−*^neutrophils increased GSH:GSSG by 45% though not significantly (*p* < 0.09) (Fig. 4C). Interestingly, the increased GSH:GSSG is the same percentage of PC3 cell death seen in the co-culture suggesting that the altered glutathione synthesis in remaining PC3 is in response to neutrophil killing (Fig. 4C). To investigate the impact of neutrophils on cancer GSH earlier during the culture, we cultured neutrophils and cancer cells directly for a maximum of 3 h and measured the GSH:GSSG ratio on C42B and PC3 cells. There was an increase in PC3 GSH:GSSG as early as 3 h after culture with *Nox2*^*−/−*^ neutrophils (Fig. 4C); however, C42B cells did not demonstrate substantial GSH changes as seen with the overnight culture s. We performed the same experiment with non-metastatic LNCaP and metastatic RM1. There was little change in RM1 glutathione after neutrophil culture though its trend appeared similar to C42B; however there was a significant increase in LNCaP GSH at 3 h of culture with neutrophils independently of Nox2 expression (Supp Fig. 4A).

**Fig. 5:**
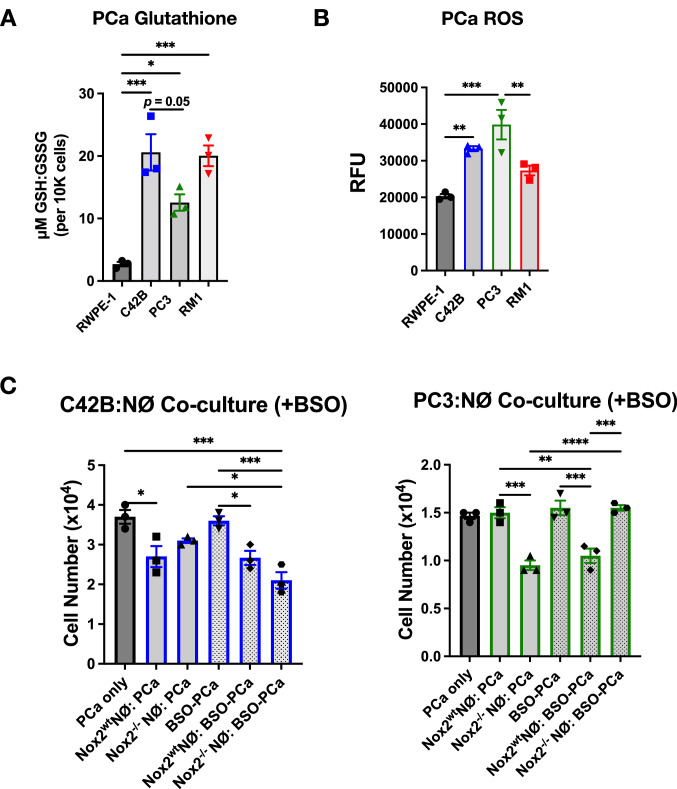
Prostate cancer glutathione synthesis impacts neutrophil-induced cell death. **A** Glutathione assay of C42B, PC3 and RM1 cells. Cells were plated at similar densities in a 96 well opaque plate. 48 h later, media was removed and cells were incubated in PBS with glutathione assay reagents. Cell number was assessed to normalize data. Data displayed as ratio of GSH:GSSG per 10,000 cells. **B** Amplex red assay measurement of prostate cancer extracellular H_2_O_2_. **C** Co-culture of C42B (left) and PC3 (right) with primary *Nox2*^*wt*^ and *Nox2*^*−/−*^ neutrophils. Prostate cancer cells were treated with 100uM BSO for 24 h, and BSO removed prior to adding neutrophils for 24 h. Graph depicts cancer cell counts after overnight incubation with neutrophils. Cancer cell number was quantified using Trypan Blue exclusion assay. n = 3 replicates per cell line. Data are represented as mean ± SEM. Statistical analysis per one-way or two-way ANOVA with p-values as follows: *p < 0.05, **p < 0.01, ***p < 0.001, ****p < 0.0001

To gain more insight into redox balance in BM-PCa, we examined glutathione (via GSH:GSSG assay) and extracellular H_2_O_2_ levels (via Amplex Red assay) in C42B, PC3 and RM1 for comparison to nonmalignant RWPE cells. There were comparable levels of glutathione in the PCa cells though there was ~ twofold less GSH:GSSH in PC3 compared to C42B and RM1 cells (Fig. 5A) based solely on the amount of reduced GSH. All PCa cell lines produce more baseline ROS than non-malignant RWPE (Fig. 5B). To examine other antioxidants in the PCa cells, we measured protein levels of common antioxidants: hemoxygenase (HO) 1, catalase, SOD1 and SOD2 in BM-PCa compared to nonmalignant RWPE cells and non-metastatic LNCaP. There were more antioxidants present in the PCa cells than non-malignant RWPE; however, there appeared to be slightly more catalase produced in C42B cells compared to the other PCa lines and no catalase or SOD1 produced by RM1. PC3 show the most SOD2 expression than any other PCa cells (Supp Fig. 4B). Collectively, these data suggest that PC3 exist, and possibly benefit from, conditions of oxidative stress induced by higher cellular H_2_O_2_ levels, reduced antioxidant capacity and, increased SOD2 which allows for generation and accumulation of cellular of H_2_O_2_.

Last, we wanted to determine whether cancer glutathione metabolism impacts neutrophil cytotoxicity. To do this, PCa cells were treated with buthionine sulphoximine (BSO), an inhibitor of gamma-glutamylcysteine synthetase (gamma-GCS) which lowers cellular GSH concentrations[42] for 24 h prior to the addition of *Nox2*^*wt*^ and *Nox2*^*−/−*^ neutrophils. We confirmed that the BSO sufficiently reduced GSH yet, interestingly, had no impact on cancer cell viability (Supp Fig. 4C). Inhibition of GSH is C42B had little impact on *Nox2*^*wt*^ neutrophil cytotoxicity which induced ~ 30% C42B cell death (*p* < 0.05); however, BSO treatment enhanced cytotoxicity of *Nox2*^*−/−*^ neutrophils against C42B (~ 50% cell death; *p* < 0.001) (Fig. 5C, left). Surprisingly we found that BSO treatment sensitized PC3 to wildtype Nox2 neutrophils; this is in contrast to our previous findings showing PC3 to be resistant to neutrophil-induced death. However, BSO treatment significantly reversed *Nox2*^*−/−*^-induced cell death(Fig. 5C, right). For comparison, we performed this experiment with RM1 cells which showed changes similar to the C42B cells (Supp Fig. 4D). These findings demonstrate that manipulation of cancer redox balance through glutathione metabolism determines response to neutrophil cytotoxicity.

## Discussion

In this study, we provided evidence of an intricate crosstalk of redox regulation between metastatic prostate cancer and neutrophils. We previously showed that bone marrow neutrophils are protective against prostate cancer growth in bone and that metastatic prostate cancer cells alter neutrophil function [12]. Based on conflicting evidence demonstrating a role for neutrophil oxidative burst in both anti- and pro-tumor immune responses [43], the goal of this study was to examine the role of prostate cancer-induced neutrophil oxidative burst in the immune response and function in the prostate tumor bone environment.

We, and others, have emphasized the molecular and cellular complexity of the prostate tumor-bone environment. Neutrophils generate reactive oxygen species (ROS), primarily to kill phagocytosed bacterial pathogens, via ATP-dependent NOX2 and mitochondrial (mtROS) oxidative phosphorylation [13, 25, 44]. We tested the impact of host *Nox2* depletion on bone metastatic prostate cancer growth in vivo using an intratibial metastasis model of mouse RM1 prostate cancer cells. RM1 growth was significantly inhibited in Nox2 knockout mice and, based on L-012 imaging, reduced tumor size correlated with reduced intra-tumoral ROS (Fig. 1). Although Nox2-null had little impact on RM1 growth in vitro after 24 h (Fig. 2), it is possible that either Nox2-mediated cell signaling [45] impacts RM1 growth. Further, because Nox2 is depleted from the entire host in vivo, it is possible that Nox2 deletion from other cell populations within the tumor microenvironment also contributed to tumor suppression. ROS from neutrophils and other myeloid cells significantly suppress cytotoxic T cell function (ROS) [46] and it is possible that reduced ROS in the environment, combined with a more reactive immune response, collectively could suppress tumor growth in bone.

Our *in* vitro data (Figs. 4 and 5) showed the importance of Nox2 in neutrophil-mediated cytotoxicity was dependent on regulation of the redox balance by antioxidants in the prostate cancer cells. Nox2-null TANs isolated from bone tumors, were slightly more cytotoxic to RM1 than wildtype TANs ex vivo*.* This was in contrast to in vitro co-cultures using tumor-naïve Nox2-null neutrophils which were equally cytotoxic to the wildtype neutrophils. We have shown previously that TAN function is altered throughout tumor progression [12] and it is possible that we would see a similar phenomenon in the Nox2-null cells and this will be explored in future studies. Surprisingly, wildtype Nox2 TANs, produced significantly less H_2_O_2_ than neutrophils from tumor-naïve wildtype mice, similar to Nox2-null wildtype neutrophils and TANs, suggesting that the tumor microenvironment reduces neutrophil ROS production in vivo (Fig. 1).

The link between neutrophil redox and function in the prostate tumor microenvironment is poorly understood. Others have shown that cellular oxidative stress contributes to dysregulation of both pro-oxidant (NOX enzymes) and anti-oxidant enzymes (catalase, superoxide dismutases, peroxiredoxins, and glutathione peroxidases) [47] and this could be seen in the neutrophil genomic changes in our study predominantly in redox-associated genes. In order to compensate for the high levels of oxidative stress in cells, endogenous antioxidant enzymes are necessary to maintain redox balance [48]. In addition to increased neutrophil ROS production, BM-PCa soluble factors predominantly affected NRF2-mediated gene expression associated with oxidative stress (Fig. 3). The transcription factor, NRF2, regulates expression of detoxifying antioxidant enzymes, through promoter binding at sites known as antioxidant response elements (ARE), and can be activated by dysregulation of NOX gene expression [41, 49]. Oxidative stress indicators including arginase 1, (*ARG1*) and heme-oxygenase 1, (*HMOX1*) were upregulated in C42B-treated neutrophils compared to LNCaP, indicating a state of neutrophil oxidative stress in a metastatic environment [50, 51]. Notably, CYP1A1, a cytochrome p450 enzyme necessary to detoxify xenobiotics [41], was 100-fold higher in metastatic C42B-treated neutrophils. This enzyme can also be increased in response to ROS levels and is able to be activated through aryl hydrocarbon receptor (AHR)-NRF2 interactions [52, 53]. Increased oxidative stress markers support the data which shows increased neutrophil ROS would be present in a metastatic cancer environment (Fig. 3) and gives insight into the impact of metastatic prostate cancer on neutrophil redox. There was little change in the expression of neutrophil catalase and peroxidoxins (PRNXD1, 3, 5), enzymes involved in reduction of H_2_O_2_ to water [54]; however C42B significantly increased neutrophil SOD2, which is required for dismutase of superoxide to H_2_O_2_ [54, 55] and may contribute to cancer-induced neutrophil H_2_O_2_ accumulation and secretion.

Additionally, a significant finding from neutrophil genomic analysis was altered synthesis of glutathione, the primary non-enzymatic intracellular antioxidant. Glutathione in its reduced form, GSH, detoxifies intracellular peroxide [48]. This reaction is catalyzed by glutathione peroxidases which maintain equilibrium of reduced GSH and its oxidized form GSSG, by driving the conversion of H_2_O_2_ to water (Fig. 5) [48]. GPX deficiency leads to an accumulation of H_2_O_2_ and leaves cells vulnerable to oxidative stress [56]. In our RNA-seq data, GPX1 and GPX4 were reduced in neutrophils incubated in metastatic cancer CM, suggesting neutrophils in a metastatic environment cannot regulate H_2_O_2_ levels. Based on evidence of the dependence of metastatic prostate cancer on ROS [57, 58], this phenomenon could be a prostate cancer-mediated mechanism to maintain ROS levels in the tumor microenvironment, though this will need to be examined further. Other genes were involved in glutathione maintenance were altered including: glutathione transferase, (GSTO1), glutathione synthetase, (GSS) and glutathione reductase, (GSR) in C42B-treated neutrophils (Fig. 3). Glucose-6-phosphatase dehydrogenase (G6PD) is an antioxidant enzyme that is the primary source of NADPH, which is utilized by NOX2 to generate superoxide from oxygen and additionally aids glutathione reductase (GR) in recycling oxidized GSSG back to its reduced form, GSH [59, 60]. An increase in G6PD in the C42B incubated neutrophils suggests an attempt to reduce ROS accumulation by increasing reduction of GSH. Of significant importance is the enzyme glutamate-cysteine ligase-catalytic subunit, GCLC, which catalyzes the first rate-limiting step of glutathione from amino acids glutamate and cysteine. Synthesis of GSH can be induced by growth factors, cytokines or hormones through enhanced GCLC transcription [61]. The higher expression of this gene in C42B-incubated neutrophils suggests a compensation for lack of intracellular recycling of GSH through upregulation of GCLC. These data highlight cancer-mediated neutrophil redox changes that would induce neutrophil oxidative stress and promote accumulation of ROS in the surrounding microenvironment.

Genetic alterations in neutrophil glutathione metabolism translated into regulation of glutathione (Fig. 4); however, these changes were only impacted by Nox2 expression in C42B-treated neutrophils. In contrast to C42B, PC3 and RM1 factors significantly reduced the GSH:GSSG ratio in neutrophils. Although the mechanisms behind this phenomenon are unclear, PC3 and RM1 both demonstrate heightened Myc activation which is associated with progression of castration-resistant prostate cancer (CRPC) and emergence of neuroendocrine prostate cancer [62–65] and this evidence could specific neutrophil regulation by aggressive prostate cancer subtypes [63, 66, 67] that promotes an imbalance of ROS in the surrounding microenvironment.

It remains unclear how oxidative versus reductive stress contributes to BM-PCa progression and survival, however, our findings demonstrate that understanding these dynamics is important to neutrophil-mediated immune response. There is significant evidence that androgens promote adaptation to oxidative stress [68] and our data showing heightened ROS levels in BM-PCa (i.e., micromolar range H_2_O_2_ production) (Fig. 5) verifies those previous studies. We found differential expression of other antioxidants in PCa compared to non-malignant RWPE prostate epithelial cells (Supp. Figure 4) suggesting a mechanism to prevent oxidative stress. However, there was noticeably lower baseline GSH in PC3 compared to the other PCa cells (LNCaP, C42B, RM1) and, additionally, PC3 express more SOD2, a primary generator of H2O2 which has also been shown to promote metastasis in other cancers [69]. Based on data with BSO inhibitors, this data suggests metabolic differences between PC3 and C42B that are important in their sensitivity to neutrophil killing.

Although oxidative stress has been associated with progression of several malignancies, reductive stress, or excessive antioxidant production, has been linked to tumor cell survival [70–72]. Based on evidence of PCa-regulated glutathione in neutrophils, we examined glutathione levels in PCa after being cultured with neutrophils. There was an increase in the ratio of glutathione in C42B cells in response to wildtype neutrophils and a further increase after culture with Nox2-null neutrophils as early as 3 h of direct contact (Fig. 4, Supp. Figure 4) suggesting a tip in balance to reductive stress, though this did not appear to be directly associated with neutrophil induced-cell death. Neutrophil-directed glutathione levels in LNCaP and RM1 cells were similar to C42B (Supp Fig. 4) and may be related to androgen sensitivity due to their androgen receptor status. In comparison, there was no change in glutathione levels in PC3 cultured with wildtype neutrophils; however, there was an increase in PC3 glutathione in response to Nox2-null neutrophils, which also PC3 cell death. Further, we found that suppression of glutathione by BSO induced PC3 cell death in response to neutrophils; yet this trend was reversed with the added ROS depletion (seen after culture with Nox2-null neutrophils). These findings suggest that PC3 oxidative stress maintains growth while regulation of reductive stress via glutathione metabolism is toxic to the cells. Freitas et al. previously showed that PC3 and other aggressive prostate cancer cells, show acquired resistance to H_2_O_2_ and that altered antioxidant capacity impacts growth [73] and was somewhat dependent on androgen sensitivity. Although RM1 cells do express AR, others have shown that RM1 are insensitive to androgen deprivation in vitro and in vivo [74, 75], thus representing aggressive prostate cancer. Our in vitro co-cultures also revealed that depletion of ROS could induce cell death of AR-negative prostate cancer cells (demonstrated in PC3 and PAIII) demonstrating that PCa sensitivity to neutrophil response is highly dependent on the balance of oxidative and reductive stress, which may be a readout for disease stage. Future experiments will focus on the relationship between neutrophil-induced cell death and glutathione metabolism in androgen-sensitive and -insensitive PCa.

In summary, we have identified that redox communication between neutrophils and PCa crosstalk in the tumor-bone microenvironment may be integral to neutrophil control of BM-PCa progression. We previously showed that neutrophils induce BM-PCa death. Here we show that, in tandem, PCa regulates neutrophil redox and intracellular redox via glutathione regulation, which determines sensitivity to neutrophil cytotoxicity. Collectively, these findings reveal that BM-PCa, and particularly aggressive AR-negative cells which would be resistant to androgen deprivation therapy, could be targeted by the combined suppression of ROS and glutathione metabolism as a novel therapeutic strategy for treating BM-PCa patients.

## Supplementary Information

Below is the link to the electronic supplementary material.

Supplementary file1 (PDF 2335 kb)

## Data Availability

The datasets generated during and/or analysed during the current study are available from the corresponding author on reasonable request. All data has been deposited in the NCBI GEO Database under Accession Number: GSE193468; data will be made available to the public January 2024.
