# Supplementary material for: Prostate cancer addiction to oxidative stress defines sensitivity to anti-tumor neutrophils
Source: Clin Exp Metastasis. 2022 May 23;39(4):641–59. doi: 10.1007/s10585-022-10170-x (PMC9338904; doi:10.1007/s10585-022-10170-x)
Supplement: Supplementary file 1 — Supplementary file1 (PDF 2335 kb) [file 10585_2022_10170_MOESM1_ESM.pdf]

# Supplemental Figure 1

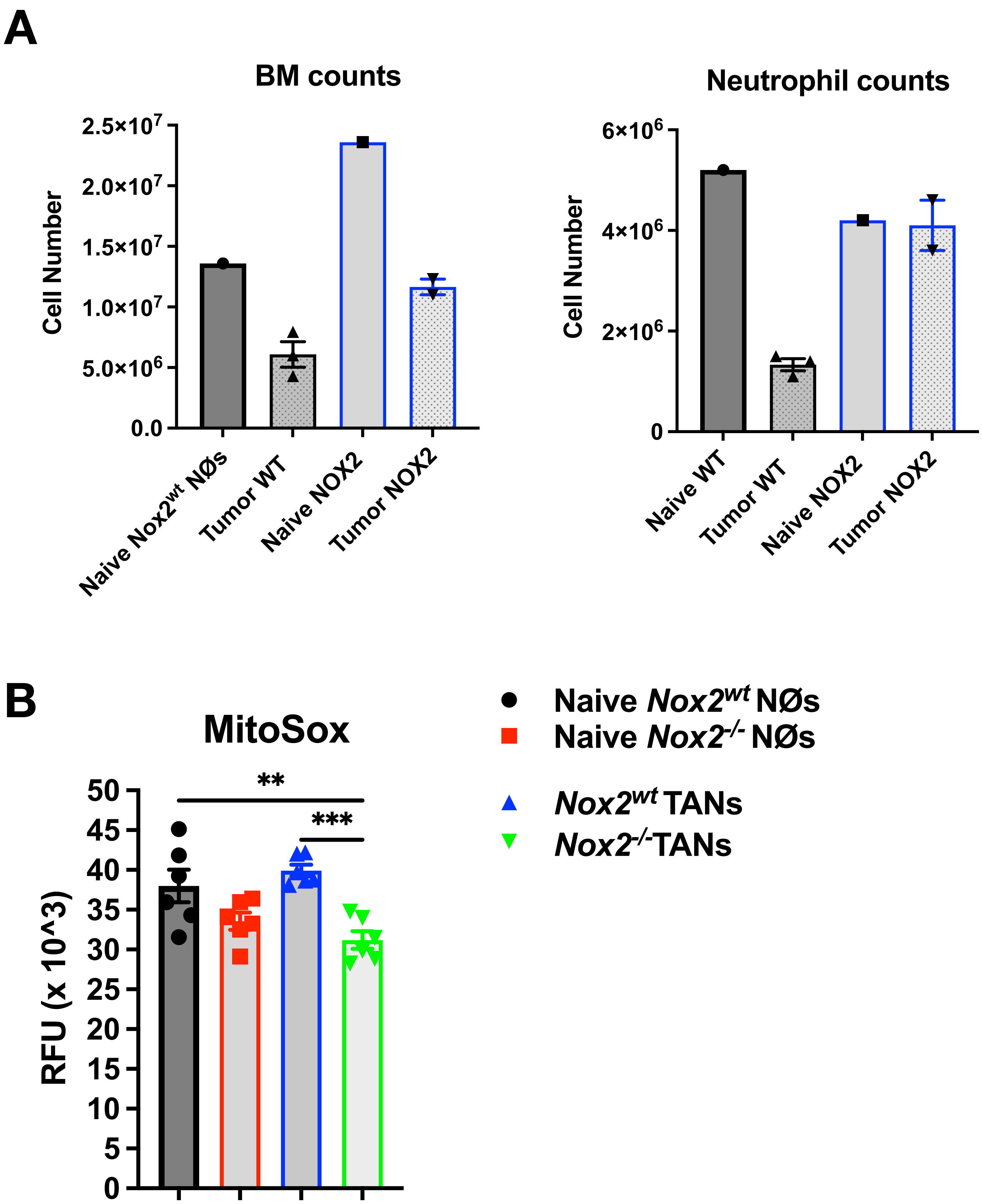


**Supplemental Figure 1. RM1 in Nox2 knockout mice.** (A) Bone marrow (BM) and neutrophil cell numbers from mouse tibia; *Nox2$^{wt}$* (n=3) and *Nox2$^{-/-}$* (n=2), graphs show average number of bone marrow and neutrophils per group. (C) MitoSox in tumor naïve neutrophils and TANs from *Nox2$^{wt}$* and *Nox2$^{-/-}$* mice; n=3 mice per group were plated in biological triplicate per experiment. Data are represented as mean ± SEM. Statistical analysis per two-sample T-test or one way ANOVA with p-values as follows: ****p*<0.001.

# Supplemental Figure 2

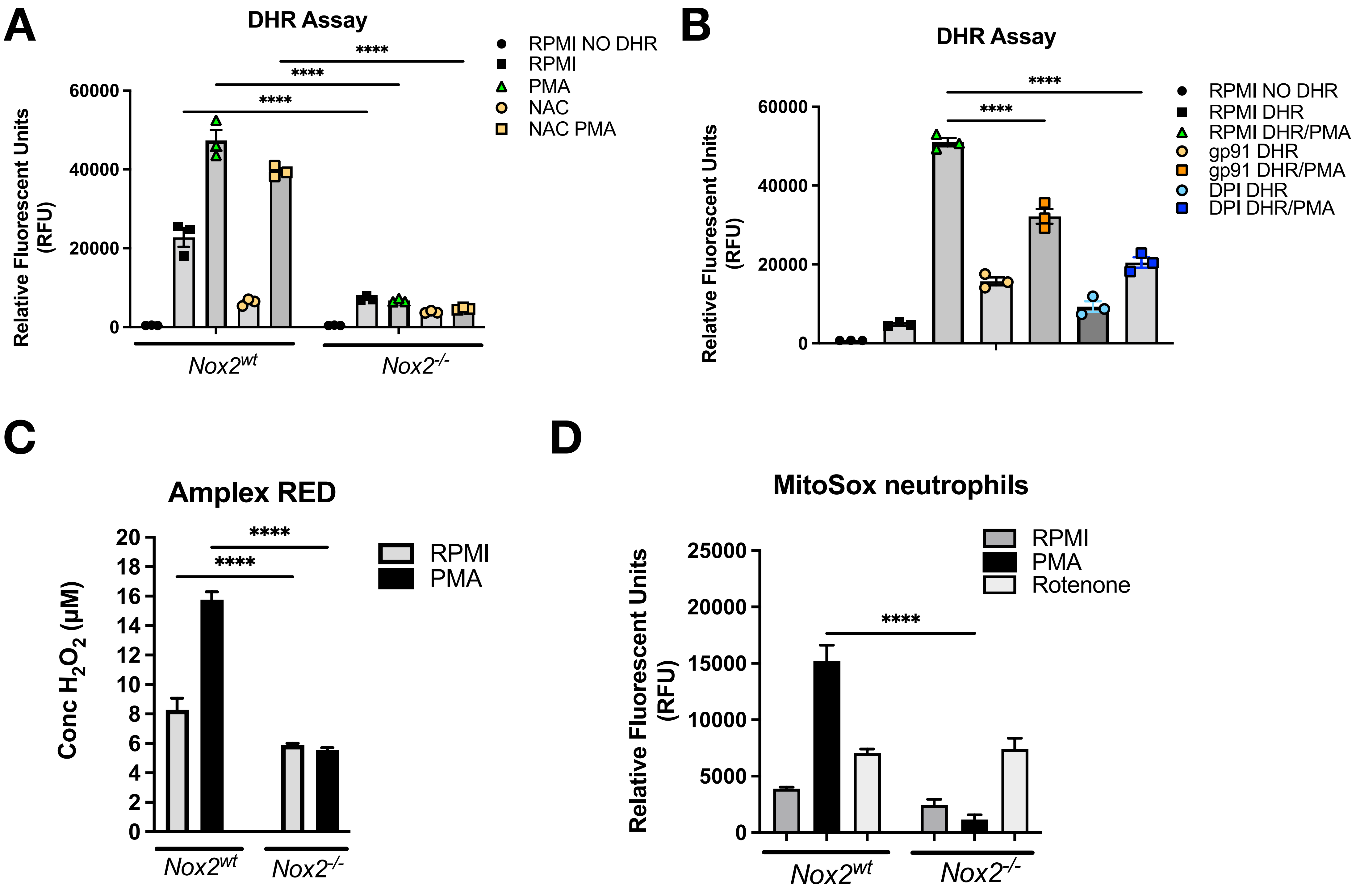


**Supplemental Figure 2. Genetic deletion of Nox2 suppresses neutrophil ROS generation.** (A) Dihydrorhodamine (DHR) assay in *$Nox2^{wt}$* and *$Nox2^{-/-}$* neutrophils, stimulated with PMA (100nM) in the presence or absence of ROS scavenger NAC (5mM) (A) or wildtype neutrophils treated with Nox2 inhibitor, gp91-ds-tat, or pan-Nox inhibitor, diphenyleneiodonium (DPI;10µM) in combination with PMA stimulation (B). Intracellular ROS is quantified via relative fluorescence of green fluorescence rhodamine in neutrophils. (C) Amplex Red assay for in *$Nox2^{wt}$* and *$Nox2^{-/-}$* neutrophils, with and without PMA (100nM) stimulation, measuring the concentration of extracellular $H_2O_2$. (D) MitoSox assay, for measurement of mitochondrial ROS of *$Nox2^{wt}$* and *$Nox2^{-/-}$* neutrophils treated with with electron transport chain (ETC) inhibitor rotenone (5µM) or PMA (100nm); n=3 each per condition. Data are represented as mean ± SEM. Statistical analysis per one-way or two-way ANOVA with p-values as follows: **p*<0.05,** *p*<0.01 ,****p*<0.001, *****p*<0.0001.

# Supplemental Figure 3

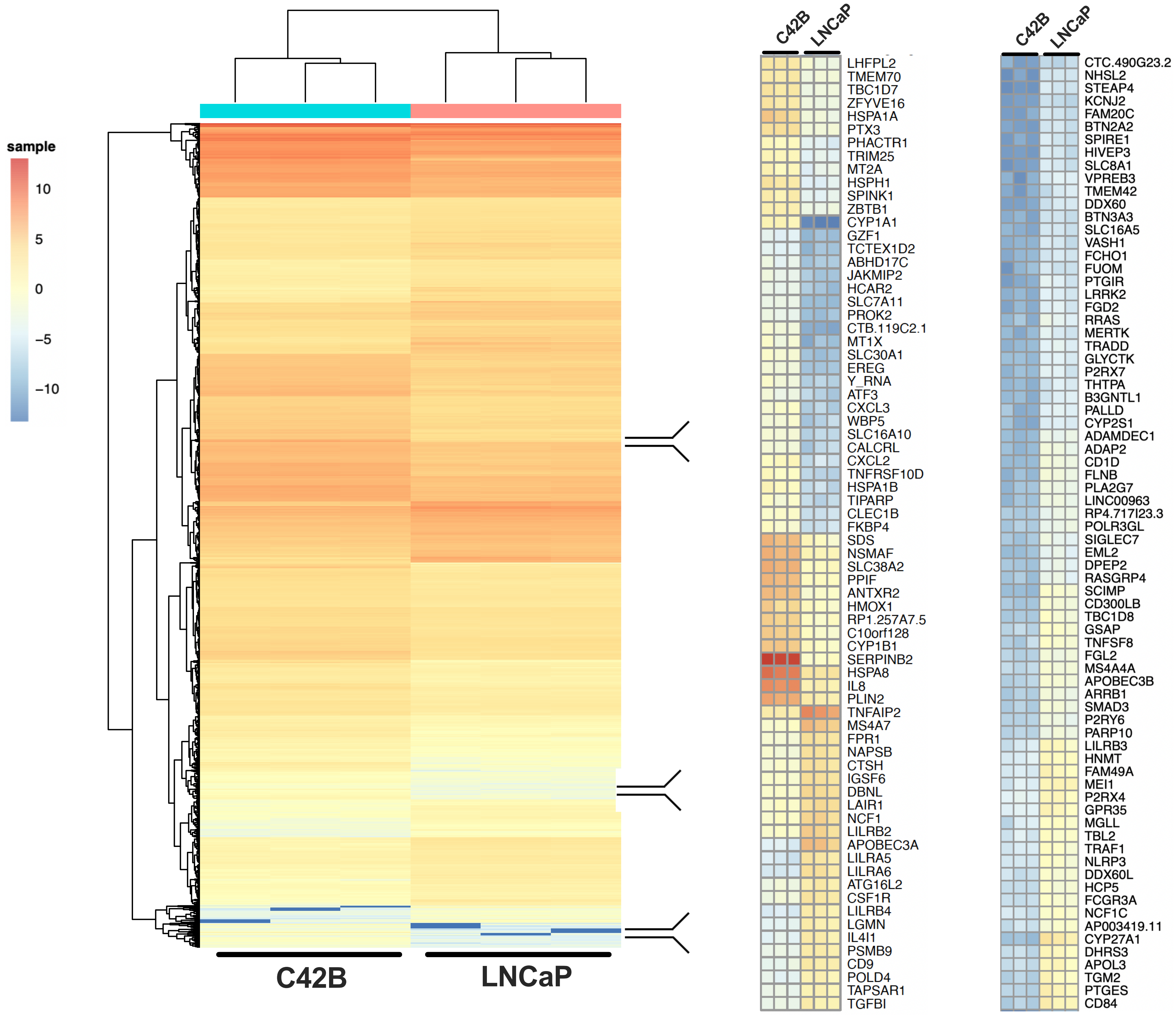


**Supplemental Figure 3. BM-PCa regulation of the transcriptome of bone marrow-derived neutrophils.** (A) Heatmap of bulk RNA sequencing of human bone marrow neutrophils treated with serum-free conditioned media from LNCaP and C42B for 3 hours. Color key; red shows increased gene expression per fold-change, blue represents reduced gene expression fold-change.

# Supplemental Figure 4

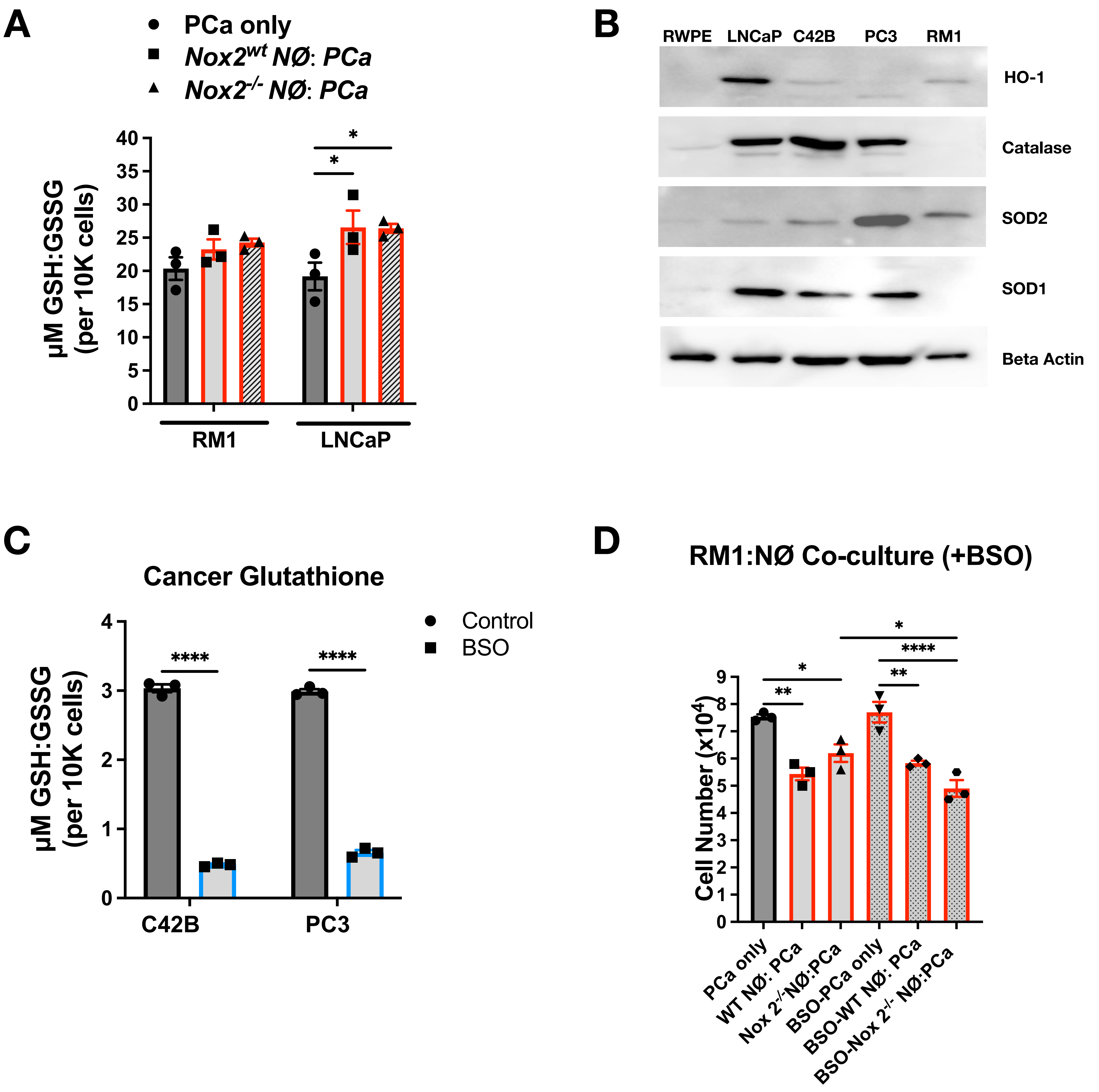


**Supplemental Figure 4. Neutrophil regulation of glutathione on cancer cell death.** (A) Glutathione assay of RM1 and LNCaP cells after 3 hour co-culture with neutrophils. Data shown per 10,000 cells as ratio of reduced GSH to oxidized GSSG. (B) Western blot of antioxidants in prostate cancer cell lines (LNCaP, C42B, PC3, RM1) and non-malignant RWPE cells. Beta actin is used as loading control. (C) Glutathione assay, shown as GSH:GSSG in C42B and PC3 and 24 hour treatment with BSO (100uM). (D) Overnight co-culture of primary bone marrow neutrophils with RM1; RM1 were treated with BSO for 24 hours prior to addition of neutrophils; graph shows Trypan Blue exclusion assay quantitation of remaining cancer cells after overnight culture with neutrophils. N=3 for each cell line. Data are represented as mean ± SEM. Statistical analysis per one-way or two-way ANOVA; p-values as follows; *$p$<0.05, **$p$<0.01, ****$p$<0.0001
